# AB569, a non-toxic combination of acidified nitrite and EDTA, is effective at killing the notorious Iraq/Afghanistan combat wound pathogens, multi-drug resistant *Acinetobacter baumannii* and *Acinetobacter spp*.

**DOI:** 10.1371/journal.pone.0247513

**Published:** 2021-03-03

**Authors:** Amy L. Bogue, Warunya Panmanee, Cameron T. McDaniel, Joel E. Mortensen, Edwin Kamau, Luis A. Actis, Jay A. Johannigman, Michael J. Schurr, Latha Satish, Nalinikanth Kotagiri, Daniel J. Hassett

**Affiliations:** 1 Department of Molecular Genetics, Biochemistry and Microbiology, University of Cincinnati College of Medicine, Cincinnati, OH, United States of America; 2 Wright-Patterson Air Force Base, Dayton (Wright-Patterson Air Force Base), Dayton, OH, United States of America; 3 Diagnostic Infectious Disease Testing Laboratory and Department of Pediatrics, Cincinnati Children’s Hospital Medical Center, Cincinnati, OH, United States of America; 4 Walter Reed National Military Medical Center (WRNMMC), Bethesda, MD, United States of America; 5 Department of Microbiology, Miami University, Oxford, OH, United States of America; 6 U.S. Army Institute of Surgical Research, San Antonio, TX, United States of America; 7 Department of Immunology and Microbiology, University of Colorado Anschutz School of Medicine, Denver, CO, United States of America; 8 Department of Pathology and Laboratory Medicine, University of Cincinnati College of Medicine, Cincinnati, OH, United States of America; 9 College of Pharmacy, University of Cincinnati College of Medicine, Cincinnati, OH, United States of America; 10 Research Department, Shriners Hospitals for Children- Cincinnati, Cincinnati, OH, United States of America; Northwestern University Feinberg School of Medicine, UNITED STATES

## Abstract

Multi-drug resistant (MDR) *Acinetobacter baumannii* (*Ab*) and *Acinetobacter spp*. present monumental global health challenges. These organisms represent model Gram-negative pathogens with known antibiotic resistance and biofilm-forming properties. Herein, a novel, nontoxic biocide, AB569, consisting of acidified nitrite (A-NO_2_^-^) and ethylenediaminetetraacetic acid (EDTA), demonstrated bactericidal activity against all *Ab* and *Acinetobacter* spp. strains, respectively. Average **f**ractional **i**nhibitory **c**oncentrations (FICs) of 0.25 mM EDTA plus 4 mM A-NO_2_^-^ were observed across several clinical reference and multiple combat wound isolates from the Iraq/Afghanistan wars. Importantly, toxicity testing on human dermal fibroblasts (HDFa) revealed an upper toxicity limit of 3 mM EDTA plus 64 mM A-NO_2_^-^, and thus are in the therapeutic range for effective *Ab* and *Acinetobacter spp*. treatment. Following treatment of *Ab* strain ATCC 19606 with AB569, quantitative PCR analysis of selected genes products to be responsive to AB569 revealed up-regulation of iron regulated genes involved in siderophore production, siderophore biosynthesis non-ribosomal peptide synthetase module (*SBNRPSM*), and siderophore biosynthesis protein monooxygenase (*SBPM*) when compared to untreated organisms. Taken together, treating *Ab* infections with AB569 at inhibitory concentrations reveals the potential clinical application of preventing *Ab* from gaining an early growth advantage during infection followed by extensive bactericidal activity upon subsequent exposures.

## Introduction

*Acinetobacter baumannii* (*Ab*) infections have historically been a major clinical challenge for both military and civilian health professionals, especially during the Iraq/Afghanistan conflicts. In the context of combat medical care and acquired wound, burn, blast and ventilator-associated pneumonia (VAP) infections, *Ab* represents a formidable multi-drug resistant (MDR-AB) pathogen and, as such, is a top 10 CDC priority organisms [1, 2]. Joint Program Committee 2, Military Infectious Disease Research Program (JPC-2/MIDRP) is a congressionally directed committee with a charter to research wound infection prevention as well as antimicrobial countermeasures. The focus research area for JPC-2/MIDRP includes poly-trauma and blast wound injuries [3, 4]. *Ab*, a common blast wound pathogen, is recognized as a nosocomial isolate that readily acquires resistance genes that especially plagues immunocompromised patients. In a retrospective study of wounded combat evacuees in 2003, Petersen et al. [5] found that of 56 patients that acquired infections, 84% were infections resulting from blast wound type injuries. Furthermore, 36% of the infections were caused by *Ab*, making it the predominant wound pathogen. Such organisms were also resistant to 80% of tested drugs [5], colloquially designating *Ab* as “Iraqibacter, [6]”. The 2003 and 2004 *Acinetobacter baumannii-calcoaceticus* complex (*ABC*) outbreak among U.S. military personnel treated in Military Treatment Facilities was investigated and discovered that 7 of 7 military field hospitals sampled recovered *ABC* strains [7]. This discovery further highlights the importance of *Ab* as a significant nosocomial pathogen as well as a top-priority, “serious threat” according to the CDC (https://www.cdc.gov/drugresistance/biggest_threats.html). In addition, mortality rates for nosocomial infections with multi-drug resistant *Acinetobacter spp*. were 26% higher than the mortality rate of uninfected patients [8]. Further compounding this problem is the fact that *Ab* possesses genomic resistance islands (***Ab* a**ntibiotic **r**esistance, AbaR) enabling the bacteria to rapidly acquire drug antibiotic resistance [9]. Furthermore, this bacterium is also naturally competent, thereby being highly proficient at the uptake of stable plasmids [10]. These characteristics make *Ab* and *Acinetobacter spp*. ideal model organisms and a huge challenge for the development of novel antibiotics or non-cytotoxic biocides, especially considering that the development of novel therapeutics has dramatically slowed in recent years.

### AB569: A novel, two component formulation for the treatment of *Ab* infections

#### i. The EDTA component

AB569 is a non-toxic, bactericidal combination of EDTA and acidified nitrite (herein A-NO_2_^-^) buffered to pH of 5.5 to 6.5. The EDTA component of AB569 is widely known as a chelator of di/tri-valent cations with a binding preference prioritizing iron (Fe^2/3+^), calcium (Ca^2+^), and magnesium (Mg^2+^) [11]. EDTA also increases the permeability of the bacterial cell wall by binding Ca^2+^ and Mg^2+^ ions that bridge the vital lipopolysaccharide (LPS) component within the outer membrane of Gram-negative bacteria [12]. The ability of EDTA to also chelate metals, especially Fe, dramatically influences the ability of *Ab* to form complex, antibiotic-resistant, highly organized communities known as biofilms [13]. Biofilm formation by *Ab* is considered a significant virulence property, thereby enhancing its ability to cause disease [14]. Fe also influences the robustness of *Ab* biofilm formation [15] and we speculate that its sequestration may play a translational role in future clinical studies designed to treat highly problematic *Ab* and *Acinetobacter spp*. infections. Taken together, the role of EDTA in altering the permeability of bacterial cell walls, its ability to sequester Fe, and its effect on biofilm formation are just a few of the actions that contribute to the bactericidal status of EDTA that has been observed in multiple bacterial pathogens [16–18].

#### ii. The acidified nitrite (A-NO_2_^-^) component

Bacteria can also be exposed to potentially toxic doses of reactive nitrogen species (RNS) during the course of human infection [19]. The endogenous metabolic production of or exposure to exogenous RNS has been shown to enable bacteria to acquire resistance to some antibiotics [20]. Treatment of bacteria with A-NO_2_^-^ has been shown to increase RNS formation in the form of nitric oxide (NO) production [21]. Given its ability to diffuse through bacterial cell membranes [22], NO can potentially combine with the one-electron reduction product of molecular oxygen, superoxide (O_2_^-^), to form peroxynitrite (OONO^-^), an exceedingly powerful oxidant that can react with virtually all known biomolecules at diffusion-limited rates (~10^10^ M^-1^s^-1^, [23]). Still, the predominant means by which OONO^-^ causes cell death is thought to be predominantly due to DNA damage [24]. In addition, excess A-NO_2_^-^ can lead to lipid peroxidation and increased cell permeability or tyrosine nitration that can alter cell function, structure or homeostasis [25]. NO also binds to cysteine residues on proteins, forming S-NO proteins (via ***S***-**n**itr**o**sylation/**n**itr**o**sation), potentially inhibiting proper protein processing and overall cellular function [26].

Despite the fact that the genus *Acinetobacter* are strict aerobes, they still have the ability to reduce nitrate to nitrite using reductase enzymes [27, 28], further “recycling” A-NO_2_^-^. *Ab* also converts nitrate to ammonia and uses nitrogen as an energy source in the assimilatory nitrate reduction pathway. *Ab* is also capable of converting nitrite (NO_2_^-^) to ammonium hydroxide (NH_3_OH) via an NADH/NADPH nitrite reductase [29]. As there is likely a myriad of targets/processes adversely affected during the process of A-NO_2_^-^ mediated bacterial killing, the direct mechanism of bactericidal action, as with any biocide (e.g., HOCl, H_2_O_2_, F^-^, etc.), is unknown. Finally, in a previous study, Yoon et al. [21] showed that at slightly acidic pH levels, A-NO_2_^-^ can give rise to HNO_2_ that is unstable and spontaneously generates NO, N_2_O_3_, NO_2_^.^ and ultimately NO_3_^-^.

Though many bacteria are sensitive to A-NO_2_^-^, a previous study by McDaniel et. al. [30] showed increased sensitivity of the ESKAPE pathogen *Pseudomonas aeruginosa* (*Pa*) to AB569 compared to using either EDTA or A-NO_2_^-^ alone in a synergistic fashion. *Pa* differs from *Ab* in the metabolism of nitrate, due to its ability to grow under both aerobic and anaerobic conditions, the latter of which is by the process of denitrification. Moreover, current studies have also revealed sensitivity to AB569 in bacteria such as other ESKAPE pathogens including methicillin-resistant *Staphylococcus aureus* (MRSA) strain USA300, *Enterococcus faecium*, *Klebsiella pneumoniae* and *Enterobacter sp*., as well as *Escherichia coli* and many other Gram-positive and Gram-negative organisms [31].

The promise of AB569 as a therapeutic bacterial biocide is particularly relevant considering that bacteria are unable to develop resistance to it over time. In another combat wound/blast pathogen, *Pa*, AB569 significantly compromised >30 vital pathways including those involved in the biosynthesis of DNA, RNA, protein and ATP, leading to rapid killing of such bacteria [32]. Thus, given the interests of military and civilian practitioners in antibiotic resistance mechanisms and wound infection prevention and treatment, the goals of this study were to test AB569 against clinical isolates of *Ab* and *Acinetobacter spp*. with seven separate experimental challenges that are described in detail below. We found that all Iraq and Afghanistan battlefield clinical isolates as well as reference strains were sensitive to AB569, some of which were in a synergistic fashion. In addition, bactericidal concentrations of AB569 were non-toxic to primary adult human skin (dermal) fibroblasts. Regarding human use, both the NaNO_2_ and/or EDTA component(s) of AB569 have separately proven to be safe in studies related to the treatment of urinary tract infection [33], burn wounds [34], cystic fibrosis lung infection [35], chelation therapy [36], soaps [37] and cosmetics [38]. Thus, our results suggest that AB569 has great potential as a therapeutic agent form the treatment of battlefield wound/burn/blast infections as well as problematic ventilator-associated pneumonia (VAP) infections by *Ab*.

## Materials and methods

### Bacteria used in this study

All bacteria used in this study are listed in **Table 1**. The bacteria were maintained as frozen stocks in a 1:1 suspension of Luria-Bertani (L-broth) or Trypticase soy broth and 30% glycerol at -80°C.

**Table 1:** Bacterial strains, cell lines and primers used in this study.

Bacterial Strain, Primer, or Cell Line	Short ID	Source	Description or DNA sequence (5′ to 3′)	Source or reference
**Bacterial Strains: *Acinetobacter baumannii (Ab)* or *Acinetobacter Spp*.**				
*Ab*	*Ab*-Epi	-	Clinical Isolate	USAFSAM PHE
*A*. *iwoffi*	AI-EPI	Urine	Clinical Isolate	USAFSAM PHE
*Ab* ACICU	ACICU	-	Clinical Isolate	Miami University
*Ab* AYE	AYE	-	Clinical Isolate	Miami University
*Ab* ATCC 17978	17978	-	ATCC Strain	Miami University
*A*. *iwoffi*	WP#2	-	Clinical Isolate	88 DTS/ SGQC
*Ab*	WP#1	-	Clinical Isolate	88 DTS/ SGQC
*Acinetobacter spp*	GNR 3-9J	-	Clinical Isolate	CCHMC
*Acinetobacter spp*	GNR 3-IG	-	Clinical Isolate	CCHMC
*Acinetobacter spp*	GNR 3-2G	-	Clinical Isolate	CCHMC
*Acinetobacter spp*	GNR 3-10J	-	Clinical Isolate	CCHMC
*Acinetobacter spp* genomospecies 9 ATCC 9957	9957	-	ATCC Strain	CCHMC
*A*. *anitratus* ATCC 49139	49139	-	ATCC Strain	CCHMC
*Ab* ATCC 19606	19606-JM	-	ATCC Strain	CCHMC
*Ab* ATCC 747	747	-	ATCC Strain	CCHMC
*A*. *anitratus* ATCC 49137	49137	-	ATCC Strain	CCHMC
*Acinetobacter spp*	GNR 3-8E	-	Clinical Isolate	CCHMC
*Acinetobacter spp*	GNR 3-3C	-	Clinical Isolate	CCHMC
*Ab*	B2-MRO12	-	Clinical Isolate	CCHMC
*Ab*	B1-MRO11	-	Clinical Isolate	CCHMC
*Ab*	WR 1	Bone	Clinical Isolate	WRNMMC
*Ab*	WR 2	Urine	Clinical Isolate	WRNMMC
*Ab*	WR3	Sputum	Clinical Isolate	WRNMMC
*Ab*	WR4	Hip	Clinical Isolate	WRNMMC
*Ab*	WR5	Wound	Clinical Isolate	WRNMMC
*Ab*	WR6	Urine	Clinical Isolate	WRNMMC
*Ab*	WR7	Groin	Clinical Isolate	WRNMMC
*Ab*	WR8	Tissue	Clinical Isolate	WRNMMC
*Ab*	WR9	Tissue	Clinical Isolate	WRNMMC
*Ab*	WR10	Groin	Clinical Isolate	WRNMMC
*Ab*	WR11	Hip	Clinical Isolate	WRNMMC
*Ab*	WR12		Clinical Isolate	WRNMMC
*Acinetobacter spp*	WR13		Clinical Isolate	WRNMMC

### Bacterial identification

Bacterial identification was confirmed using the Vitek MS (bioMerieux, Marcy-l-Etoile, France)—**M**atrix **A**ssisted **L**aser **D**esorption **I**onization–**T**ime **o**f **F**light (MALDI-TOF) using and FDA cleared–IVD database following manufacturers guidelines. This database contains the following: *Acinetobacter* spp., *Acinetobacter baumannii* complex, *Acinetobacter haemolyticus*, *Acinetobacter junii*, and *Acinetobacter lwoffii*. Additional data is available for *A*. *ursingii* but is not sufficient for an IVD claim.

### Minimum Inhibitory Concentration (MIC) testing

MIC testing was performed using the Vitek 2 system (bioMerieux, Marcy-l-Etoile, France) following manufacturer guidelines. Additional MICs were determined for colistin using the Etest system (bioMerieux, Marcy-l-Etoile, France) with 150 mm cation adjusted Mueller-Hinton agar plates (BD Diagnostics, Sparks, MD) incubated at 35°C for 18 to 24 hr in ambient air, following manufacturer guidelines.

### Fractional Inhibitory Concentration (FIC) determinations

**Fig 1:**
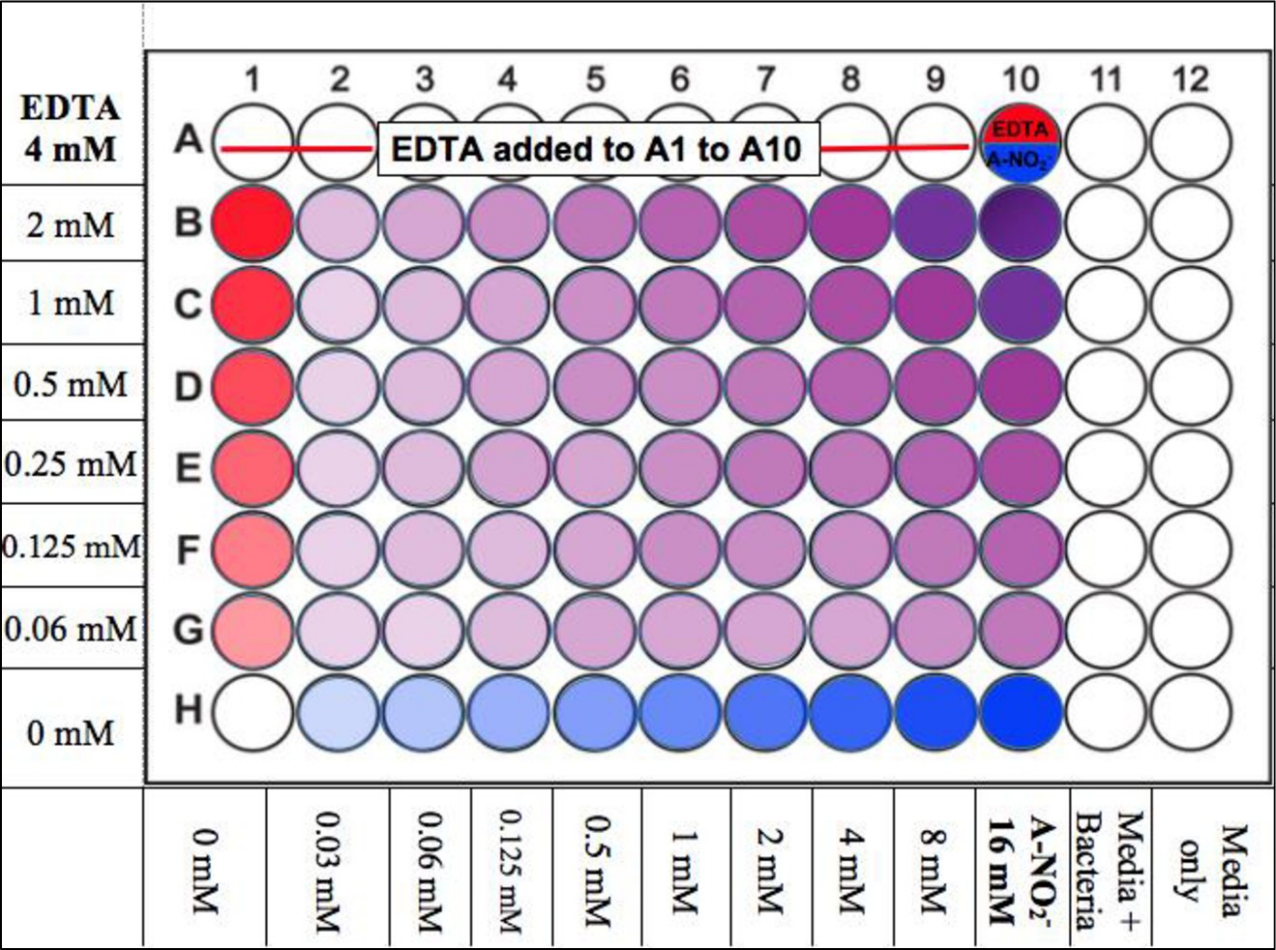
Illustration of the FIC checkerboard scheme, 96-well plate set up. The red wells are EDTA, blue wells are A-NO_2_^-^_,_ and purple wells are the AB569 combination at various concentration as indicated.

Microtiter plates (96-well) were prepared in a checkerboard scheme as previously described [39] **(****Fig 1**). Stock EDTA and freshly prepared NaNO_2_ solutions were diluted in Luria-Bertani (LB) broth plus 100 mM potassium nitrate (KNO_3_) and 50 mM potassium phosphate buffer at pH 6.5 (Kp_i_, LBN 6.5) or Trypticase Soy Broth (TSB) plus 100 mM KNO_3_ and 50 mM Kp_i_ at pH 6.5 (TSBN 6.5) at 2-fold increasing levels to final concentrations of 16 mM EDTA and 64 mM A-NO_2_^-^ and also a combination of the two. The two media used was based upon the growth preferences of each strain, as some were difficult to grow. The nitrate was added to the medium since wounded/burned murine serum has been shown to possess higher nitrate/nitrite levels [40]. Most of the bacteria used in this study were grown in LB. However, if some bacteria could not grow or grew poorly in LB, TSB was used. Bacteria were incubated at 37°C overnight in LB or TSB broth and were centrifuged at 13,000 x *g* for 2 min at room temperature. The pellet was resuspended in PBS to a final optical density (O.D._600_) of ~0.5. This suspension was then added to either LBN, 6.5 or TSBN, 6.5 at a dilution factor of 1:5000 (~5 x 10^5^ CFU/ml). Subsequently, the diluted bacterial suspension was added to all wells except for the media control column. The plates were then incubated overnight at 37°C and analyzed on the O.D._630_ channel of a Bio-Tek ELx 800 Universal Microplate Reader.

### AB569 killing studies

*Ab* ATCC 19606 was grown from a -80°C frozen stock on LB agar at 37°C overnight. A single colony was inoculated in LB broth and grown aerobically overnight at 37°C with shaking at 200 rpm. The stationary phase culture was then diluted 100-fold in LBN 6.5 and grown under the same conditions for an additional 1 hr. Treatment with EDTA, A-NO_2_^-^ or the AB569 combination at various concentrations were then added and viability of serially diluted samples evaluated over a period of 48 hr. Suspensions were diluted 10-fold in PBS and 50–100 μl aliquots were spread evenly on LB agar plates. After the plates were incubated at 37°C for 16–18 hr, bacterial colonies were enumerated and reported as Colony Forming Units (CFU) per ml.

### Quantitative polymerase chain reaction (qPCR) of *Ab* gene expression after treatment with AB569

The primers used for these experiments were based on the DNA sequence in the 470.2129 version of the *Ab* ATCC 19606 genome in the Patric.org database [41]. All primers used are listed in **Table 2**. An overnight culture of *Ab* ATCC 19606 was diluted 100-fold with LBN 6.5. Diluted bacteria were incubated for 1 hr under the same conditions used in the aforementioned killing studies. Then, bacterial cultures were treated with AB569 or its individual components for 2.5 hr under the same conditions. Bacteria were pelleted and lysed following the Qiagen RNeasy Protect Bacteria Mini Kit (Qiagen) protocol for total RNA isolation. The lysate was then pipetted into RNeasy spin columns. DNase was introduced into the column to remove contaminating DNA from the RNA samples. After non-specific binding components were washed from the column, total RNA was then eluted using RNase-free water. Prior to qPCR analyses, total RNA samples were examined for contaminating DNA by PCR using ChoiceTaq Mastermix and total RNA as a template to amplify a known housekeeping gene, *rpoD* [42]. The concentration of DNA-free total RNA sample was next analyzed using the Thermo Scientific Nanodrop method. Total RNA samples were then reverse-transcribed to cDNA using the ImProm-II^TM^ Reverse Transcription System by following the provided protocol. The cDNA was then used as a template to perform qPCR using a StepOnePlus System (Thermo Scientific, Applied Biosystems) analyzer with PowerUp SYBR Green Master Mix (Applied Biosystems) to amplify the genes of interest. To standardize the assay, 2^-ΔΔCt^ values were calculated and normalized to *rpoD* [42], annotated in **Table 2**, using the raw cycle threshold (Ct) values (also known as cross point (Cp) values) generated from our analyses [43, 44].

**Table 2:** Primers used in this study.

*Ab*	S1 Mutant	*Ab* ATCC 19606 acinetobactin siderophore mutant	Miami University
**Primers**			
RNA polymerase sigma factor *rpoD* (Housekeeper Gene)	*rpoD*	Forward **CCGATCAGGCTCGTACAATTC** Reverse **CACGGCCCATTTCCTGTAATA**	IDT
Pyruvate dehydrogenase E1 component	*PDHcE1*	Forward **GCATGTTCCAATTCAACGTCTC** Reverse **ACGAATACGGCGTTCCATATC**	IDT
LSU ribosomal protein L4p (L1e)	*L4p*	Forward **CTCTGCTGTTGAATTGTCTGAAG** Reverse **GACGACCACCTGCTAAGTAAG**	IDT
SSU ribosomal protein S11p	*S11p*	Forward **CGGCTTTGGATTACGGTTTG** Reverse **TATAACCCACTGCGCCTAATG**	IDT
Siderophore biosynthesis non-ribosomal peptide synthetase modules	*SBNRPSM*	Forward **CCGACGTCCGGCATATTATT** Reverse **TCGTCTGTTGTAGCGTGTTT**	IDT
Siderophore biosynthesis protein, monooxygenase	*SBPM*	Forward **TCGTTGGATCACACGTTCTG** Reverse **CTCTAGGCAGGCTTTGGAAATA**	IDT
SSU ribosomal protein S2p	*S2p*	Forward **GATGCTTTGAACTTCGCTAACC** Reverse **AGCTTGTTCACGGATGATGT**	IDT
Translation elongation factor Ts	*EFTs*	Forward **AGGCGAACAATTGGCTATCT** Reverse **ACGTGCATTGCAATACCTTTAC**	IDT
SSU ribosomal protein S7p	*S7p*	Forward **GTGAGATCCTTCCAGATCCTAAAT** Reverse **CCGTAAACGATACTTTCAGCAATAG**	IDT
LSU ribosomal protein L28p zinc-independent	*L28p*	Forward **TCTCACACGCCAACAACAA** Reverse **AGTGGTTAAACGAAGACGTACAA**	IDT
ATP synthase delta chain	*atpD*	Forward **AGCAAGGTGCAACAGACA** Reverse **GGAGTAAGTTCAGGGCGATTTA**	IDT

### AB569 biofilm inhibition testing

Experiment were performed in 96-well plates as described for the FIC studies above. After an overnight incubation at 37°C, the bacteria biofilms were first washed twice with PBS and stained with filter-sterilized 0.1% crystal violet. After a 30 min incubation at room temperature, unattached bacteria were removed gently by rinsing with water. Ethanol (95%) was then added to each well to dissolve the crystal violet that was bound to the peptidoglycan layer of biofilm bacteria [45]. The quantity of solubilized crystal violet was then measured at O.D._595_ using Bio-Tek ELx 800 Universal Microplate Reader.

### AB569 biofilm dispersion testing

An overnight culture of *Ab* in LB or TSB broth was diluted 100-fold into the same media and 200 μl of diluted bacteria culture was transferred into each well in 96-well plates and incubated at 37°C for 24 hr without shaking to allow the bacteria biofilms to mature. After unattached, planktonic bacteria were removed and the biofilms washed with LBN or TSBN, pH 6.5, media containing 2-fold dilutions of EDTA, A-NO_2_^-^ or AB569 was transferred into the plates. The plates were then incubated under the same conditions as above. After 24 h, the optical density of the 96-well plate suspensions was measured on the O.D._630 nm_ channel using a Bio-Tek ELx 800 Universal Microplate Reader. This was followed by the crystal violet biofilm staining assay as described above.

### Cell culture

Adult Human Dermal Fibroblast (HDFa) cells (ThermoFisher Scientific, Waltham, MA, Cat# CO135C) were cultured in DMEM supplemented with 5% fetal bovine serum (FBS), 5 μg/ml insulin, 10 ng/ml epidermal growth factor (EGF), 0.5 μg/ml hydrocortisone, and 1% penicillin streptomycin. Cells were grown in 150 mm plates in 20 ml of supplemented media with a typical dilution of 1:5 or 1:10 to promote uniform cell growth.

### SYTOX® orange dead cell staining

HDFa cells were plated at 5 x 10^4^ or 1 x 10^5^ cells per well in 12- or 24-well plates, respectively. After an overnight incubation at 37°C in an atmosphere of 5% CO_2_, medium was aspirated and the cells were treated with varying concentrations of EDTA and/or A-NO_2_^-^ in supplemented DMEM media which was acidified with 50 mM KP_i_, pH 6.5 (supplemented DMEM 6.5). Control and treated HDFa cells were incubated under the same conditions for 24 hr. To quantify the dead cells, the media from each well was transferred to individual microcentrifuge tubes. **D**ulbecco’s **P**hosphate **B**uffered **S**aline (DPBS) was used to rinse the cells in each well followed by the addition of 0.05% trypsin. The plates were subsequently incubated for 30 min at 37°C in an atmosphere of 5% CO_2_. Next, the removed media was used to neutralize the trypsin and control and treated HDFa cells were centrifuged for 5 min at 500 *x g*. The cell pelleted was re-suspended in 250 or 500 μl of supplemented DMEM media containing the Sytox Orange dead cell stain at a ratio of 1:1000 at a final concentration of 250 nM. After incubation at room temperature for 30 min, the live/dead cell ratio was analyzed by flow cytometry (Becton Dickinson (BD) FACS Calibur analysis). Stained cells were excited at 488 nm and read on the orange FL2 channel at an emission wavelength of 570 nm. Results were reported as the percentage of dead cells.

### Statistical analyses

Statisitical analyses were performed using GraphPad Prism 7 software. Statisitical significance was set at a *p*-value of <0.05.

## Results

### Bacterial identification and antibiotic minimum inhibitory concentration (MIC) determinations

*Ab* and *Acinetobacter spp*. are aerobic, oxidase-negative, Gram-negative bacilli that possess the ability to form highly problematic biofilms during disease [13]. As previously mentioned, *Ab* is known for easily acquiring multi-drug resistance during treatment of infected patients. The ensemble of bacteria used in this study included 25 *Ab* and 8 *Acinetobacter spp*. strains, including 13 MDR clinical strains provided by Walter Reed Army Medical Center (WRAMC) and 11 other clinical strains from other sources that are listed in **Table 1**. MALDI-TOF was used to identify each strain and our results showed 6/8 were *A*. *ursingii*, 1/8 was *A*. *radioresistens*, and 1/8 was *A*. *iwoffii*, respectively (**Table 1**). Antibiotic sensitivities as a collective result of minimum inhibitory concentration (MIC) testing and bacterial identification analysis were performed on each isolate. Our MIC results showed that all of the bacterial isolates used in this study were found to be resistant to cefazolin (CFZ), a first-generation cephalosporin (**Table 3**), consistent with *Ab* being a notorious β-lactamase producer [46] and also with previous studies [47, 48]. In addition, none of the bacteria tested were resistant to colistin (CST, polymyxin E) an antibiotic with high human toxicity and, as a result, a last choice for the treatment of MDR organisms [49]. According to current clinical standards of antibiotic selection by physicians for *Ab* treatment [50], meropenem (MEM) is typically the first choice in *Ab* antibiotic therapy. We found that the strains used in this study were 37% resistant to MEM (**Table 3**). Second-tier antibiotics used in this study revealed lower resistance with tobramycin (TOB, 23%) and gentamicin (GEN, 39%), respectively. It is worth noting that the 25 clinical isolates in this study meet the definition of Multi-Drug Resistant Organisms (MDRO) in that each strain was resistant to more than one class of antibiotics (**Table 3**). The percentage of antibiotic resistance for each organism was 33% for *Aa*, 0–43% for *Au* strains, 0–7% for *Ai* and ~14% for *Ar*, while *ABC* strains revealed far higher antibiotic resistance, reaching an average of 66% (range of 34–94%), respectively (**Table 3**).

**Table 3:** Antibiotic minimum inhibition concentration (MIC) and bacterial identification results.

Strain	ID by MALDI-TOF	AMP	SAM	TZP	CFZ	FOX	CAZ	CRO	FEP	MEM	GEN	TOB	CIP	NIT	SXT	CST
**Bacterial Strains: *Acinetobacter baumannii* (*AB*) or *Acinetobacter Spp*.**																
**Percent Resistance**		**88.6%**	**31.4%**	**64.3%**	**100.0%**	**94.3%**	**80.0%**	**94.3%**	**65.7%**	**37.1%**	**38.9%**	**22.9%**	**51.4%**	**97.1%**	**57.1%**	**0.0%**
*Ab*-Epi	*ABC*	≥32	≥32	≥128	≥64	≥64	4	16	≥64	≥16	≤1	≤1	≥4	≥512	≤20	0.38
		R	R	R	R	R	R	I	R	R	S	S	R	R	S	S
*AI*-EPI	*AI*	*	*	*	*	*	*	*	*	*	≤1	*	*	*	*	0.094
		*	*	*	*	*	*	*	*	*	S	*	*	*	*	S
ACICU	*ABC*	≥32	16	≥128	≥64	≥64	≥64	≥64	≥64	≥16	4	8	≥4	≥512	≥320	0.38
		R	I	R	R	R	R	R	R	R	S	I	R	R	R	S
AYE	*ABC*	≥32	4	≥128	≥64	≥64	≥64	≥64	≥64	1	≥16	≥16	≥4	≥512	≥320	0.38
		R	S	R	R	R	R	R	R	S	R	R	R	R	R	S
17978	*ABC*	≥32	≤2	≤4	≥64	≥64	4	16	2	≤0.25	≤1	≤1	≤0.25	≥512	160	0.19
		R	S	S	R	R	S	I	S	S	S	S	S	R	R	S
WP#2	*ABC*	≥32	4	≥128	≥64	≥64	≥64	≥64	≥64	1	4	≤1	≥4	≥512	≤20	0.5
		R	S	R	R	R	R	R	R	S	S	S	R	R	S	S
WP#1	*ABC*	≥32	4	≥128	≥64	≥64	≥64	≥64	≥64	1	4	≤1	≥4	≥512	≤20	0.38
		R	S	R	R	R	R	R	R	S	S	S	R	R	S	S
GNR 3-9J	*AU*	16	≤2	*	≥64	≥64	32	32	8	≤0.25	≤1	≤1	0.5	256	≤20	0.125
		I	S	*	R	R	R	I	S	S	S	S	S	R	S	S
GNR 3-IG	*AU*	16	≤2	*	≥64	≥64	≥64	32	8	≤0.25	≤1	≤1	≤0.25	≥512	≤20	0.125
		I	S	*	R	R	R	I	S	S	S	S	S	R	S	S
GNR3-2G	*AU*	4	≤2	*	32	16	2	16	≤1	≤0.25	≤1	≤1	≤0.25	≥512	≤20	0.125
		S	S	*	R	I	S	I	S	S	S	S	S	R	S	S
GNR 3-10J	*AU*	8	≤2	*	≥64	≥64	16	16	2	≤0.25	≤1	≤1	≤0.25	≥512	≤20	0.125
		S	S	*	R	R	I	I	S	S	S	S	S	R	S	S
9957	*AI*	≤2	≤2	*	16	≤4	≤1	4	≤1	≤0.25	≤1	≤1	≤0.25	32	≤20	0.094
		S	S	*	I	S	S	S	S	S	S	S	S	S	S	S
49139	*ABC*	≥32	≤2	8	≥64	≥64	16	16	32	1	≥16	2	1	≥512	≥320	0.25
		R	S	S	R	R	I	I	R	S	R	S	S	R	R	S
19606	*ABC*	≥32	≤2	≤4	≥64	≥64	16	16	16	1	8	≤1	1	128	≥320	0.125
		R	S	S	R	R	I	I	I	S	I	S	S	R	R	S
747	*ABC*	16*	≤2	≤4	≥64	≥64	4	16	4	≤0.25	≤1	≤1	≤0.25	≥512	≤20	0.38
		R	S	S	R	R	S	I	S	S	S	S	S	R	S	S
49137	*ABC*	16*	≤2	≤4	≥64	32	8	16	4	≤0.25	≤1	≤1	≤0.25	128	≤20	0.38
		R	S	S	R	R	S	I	S	S	S	S	S	R	S	S
GNR 3-8E	*AU*	*	*	*	*	*	*	*	*	*	*	*	*	*	*	0.094
		*	*	*	*	*	*	*	*	*	*	*	*	*	*	S
GNR 3-3C	*AU*	16	≤2	*	≥64	≥64	16	16	8	≤0.25	≤1	≤1	≤0.25	128	≤20	0.125
		I	S	*	R	R	I	I	S	S	S	S	S	R	S	S
B2-MRO12	*ABC*	≥32	8	≥128	≥64	≥64	≥64	≥64	32	≥16	≥16	≥16	≥4	≥512	≥320	0.38
		R	S	R	R	R	R	R	R	R	R	R	R	R	R	S
B1-MRO11	*ABC*	≥32	4	≥128	≥64	≥64	≥64	≥64	32	2	≤1	≤1	≥4	≥512	≥320	0.5
		R	S	R	R	R	R	R	R	S	S	S	R	R	R	S
WR 1	*ABC*	≥32	16	≥128	≥64	≥64	≥64	≥64	≥64	≥16	≤1	2	≥4	≥512	160	0.5
		R	I	R	R	R	R	R	R	R	S	S	R	R	R	S
WR 2	*ABC*	≥32	16	≥128	≥64	≥64	≥64	≥64	≥64	≥16	≥16	≥16	≥4	≥512	160	0.75
		R	I	R	R	R	R	R	R	R	R	R	R	R	R	S
WR3	*ABC*	≥32	16	≥128	≥64	≥64	≥64	≥64	32	≥16	≥16	≥16	≥4	≥512	≥320	0.5
		R	I	R	R	R	R	R	R	R	R	R	R	R	R	S
WR4	*ABC*	≥32	≤2	8	≥64	≥64	16	16	16	4	≤1	≤1	≥4	≥512	≤20	0.25
		R	S	S	R	R	I	I	I	S	S	S	R	R	S	S
WR5	*ABC*	≥32	16	≥128	≥64	≥64	≥64	≥64	≥64	≥16	8	≥16	≥4	≥512	≥320	0.38
		R	I	R	R	R	R	R	R	R	I	R	R	R	R	S
WR6	*ABC*	≥32	4	≥128	≥64	≥64	≥64	≥64	8	≥16	4	≤1	≥4	≥512	≤20	0.38
		R	S	R	R	R	R	R	S	R	S	S	R	R	S	S
WR7	*ABC*	≥32	≥32	≥128	≥64	≥64	≥64	≥64	≥64	≥16	≥16	≥16	≥4	≥512	≥320	0.5
		R	R	R	R	R	R	R	R	R	R	R	R	R	R	S
WR8	*ABC*	≥32	≥32	≥128	≥64	≥64	≥64	≥64	≥64	≥16	≥16	≤1	≥4	≥512	≥320	0.5
		R	R	R	R	R	R	R	R	R	R	S	R	R	R	S
WR9	*ABC*	≥32	4	≥128	≥64	≥64	≥64	≥64	≥64	≥16	4	8	≥4	≥512	≥320	0.5
		R	S	R	R	R	R	R	R	R	S	I	R	R	R	S
WR10	*ABC*	≥32	≥32	≥128	≥64	≥64	≥64	≥64	≥64	≥16	≥16	≤1	≥4	≥512	≥320	0.75
		R	R	R	R	R	R	R	R	R	R	S	R	R	R	S
WR11	*ABC*	≥32	16	≥128	≥64	≥64	≥64	≥64	≥64	≥16	8	≤1	≥4	≥512	≥320	0.5
		R	I	R	R	R	R	R	R	R	I	S	R	R	R	S
WR12	*ABC*	≥32	≤2	16	≥64	≥64	8	16	8	0.5	≤1	≤1	≤0.25	≥512	≤20	0.38
		R	S	S	R	R	S	I	S	S	S	S	S	R	S	S
WR13	*AR*	4	≤2	*	≥64	8	2	8	≤1	≤0.25	≤1	≤1	≤0.25	≥512	≤20	0.125
		S	S	*	R	S	S	S	S	S	S	S	S	R	S	S
S1 Mutant	*ABC*	≥32	≤2	8	≥64	≥64	16	32	16	1	8	2	1	256	≥320	0.19
		R	S	S	R	R	I	I	I	S	I	S	S	R	R	S

**NOTE; Antibiotic interpretation:** Sensitive (S), Resistant (R), and Intermediate (I) **Antibiotic abbreviations for MICs include:** Ampicillin (AMP), ampicillin-sulbactam (SAM), piperacillin-tazobactam (TZP), cefazolin (CFZ), cefoxitin (FOX), ceftazidime (CAZ), ceftriaxone (CRO), cefepime (FEP), meropenem (MEM), gentamicin (GEN), tobramycin (TOB), ciprofloxacin (CIP), nitrofurantoin (NIT), trimethoprim-sulfamethoxazole (SXT), and E-test (ug/mL) for colistin (CST) **Bacterial ID abbreviations:** *Acinetobacter baumannii-calcoaceticus* Complex (*ABC*), *Acinetobacter iwoffii* (*AI*), *Acinetobacter radioresistens* (*AR*) *Acinetobacter ursingii* (*AU*) *Insufficient Growth or interpretation adjustment by software.

### Fractional Inhibitory Concentration Index (FICI) determinations of AB569 against *Ab* isolates

The FICI is the lowest value that is optimal for two compounds to inhibit the growth of a single bacterial isolate. A FICI value can be calculated using the MIC and FIC values for the two components of AB569 (EDTA, A-NO_2_^-^) by the following equation (Eq 1):

$$FICI=FIC_{\left(A‐NO2‐\right)}+FIC_{\left(EDTA\right)}=\left(FIC_{A‐NO2‐}\right)/\left(MIC_{A‐NO2‐}\right)+\left(FIC_{EDTA}\right)/(MIC_{EDTA})$$ (Eq 1)

**Fig 2:**
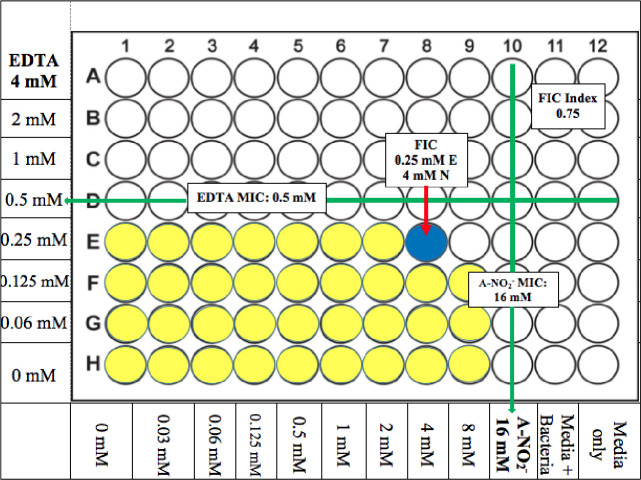
*Acinetobacter spp*. AB569 Average MIC/FIC. Average FIC index results, average FIC, and MIC concentrations. Yellow wells indicate typical bacterial growth, blue indicates well that represents FIC with inhibited bacterial growth, and white wells indicated inhibited bacterial growth. EDTA concentrations are noted on the left, A-NO_2_^-^ concentrations are annotated on the bottom, media only column 12 is listed on the bottom, and media plus bacteria column 11 is listed on the bottom. Each experiment was performed at least 3 times.

A FICI value of ≤ 0.5 indicates synergy between two compounds, whereas a FICI value of >0.5–4.0 indicates additive results and no interaction in combination. Finally, a FICI value >4 infers an antagonism by two test compounds [51]. The MIC and FICI results for all bacteria used in this study were summarized in **Table 4 and Fig 1**. Most of the FICI values of *ABC* strains used in this study were 0.56–2.25 which indicates that the majority are considered “additive” with the exception of two strains in this group (a clinical isolate from bone WR1 (WR1) and *A*. *anitratus* ATCC 49137 (49137)) which showed synergistic killing by AB569. The FICI values were 0.5 and 0.31, respectively, the same as *A*. *iwoffii* ATCC 9957 (9957), which had an FICI value of 0.375 (**Table 4 and Fig 2**). The FICI results listed in **Table 4** revealed no trends with the percentage of antibiotic resistance and also no differences between *Acinetobacter* species.

**Table 4:** AB569 Fractional Inhibitory Concentration (FIC) testing.

Bacterial Strain	EDTA MIC (mM)	A-NO_2_^-^ MIC (mM)	FIC index		EDTA FIC (mM)	A-NO_2_^-^ FIC (mM)	% Antibiotic Resistance
***Acinetobacter baumannii calcoaceticus* complex (*ABC*)**							
**WP#2**	0.25	4	2.25	0.06		8	60.00
**WR 4**	1	16	1	0.5		8	53.33
**WR 6**	0.5	16	1	0.25		8	60.00
**S1 Mutant**	0.25	16	1	0.125		8	60.00
***Ab*-Epi**	0.5	16	0.75	0.125		8	0.00
**AYE**	0.5	16	0.75	0.125		8	80.00
**WP#1**	1	16	0.75	0.5		4	60.00
**49139**	0.5	16	0.75	0.125		8	60.00
**WR 2**	1	16	0.75	0.5		4	93.33
**WR 3**	0.5	16	0.75	0.125		8	93.33
**WR 5**	1	16	0.75	0.5		4	93.33
**WR 7**	1	16	0.75	0.5		4	93.33
**WR 10**	1	16	0.75	0.5		4	86.67
**WR 11**	1	16	0.75	0.5		4	86.67
**17978**	1	16	0.75	0.25		8	40.00
**747**	1	16	0.75	0.5		4	33.33
**WR 12**	1	8	0.75	0.5		2	33.33
**19606**	0.5	16	0.625	0.06		8	60.00
**B2-MRO12**	1	16	0.625	0.125		8	86.67
**B1-MRO11**	0.5	16	0.625	0.06		8	66.67
**WR 8**	1	16	0.625	0.5		2	86.67
**WR 9**	1	16	0.625	0.5		2	80.00
**ACICU**	1	16	0.56	0.06		8	86.67
**WR 1**	0.5	16	0.5	0.125		4	80.00
**49137**	0.5	16	0.31	0.125		1	33.33
***Acinetobacter iwoffii (Ai)***							
***AI*-EPI**	0.25	8	**0.75**	0.06		4	0
**9957**	0.5	16	**0.375**	0.06		4	7.14
***Acinetobacter radioresistens (Ar)***							
**WR 13**	0.25	16	0.625	0.125		2	14.29
***Acinetobacter ursingii* (*Au*)**							
**GNR 3-IG**	0.125	8	1.5	0.06		8	42.86
**GNR 3-3C**	0.25	8	1.25	0.06		8	42.86
**GNR 3-2G**	0.25	8	1	0.125		4	28.57
**GNR 3-8E**	0.25	8	1	0.125		4	0.00
**GNR 3-10J**	0.25	16	0.75	0.06		8	35.71
**GNR 3-9J**	0.125	8	0.56	0.06		0.5	42.86

### AB569 killing studies

**Fig 3:**
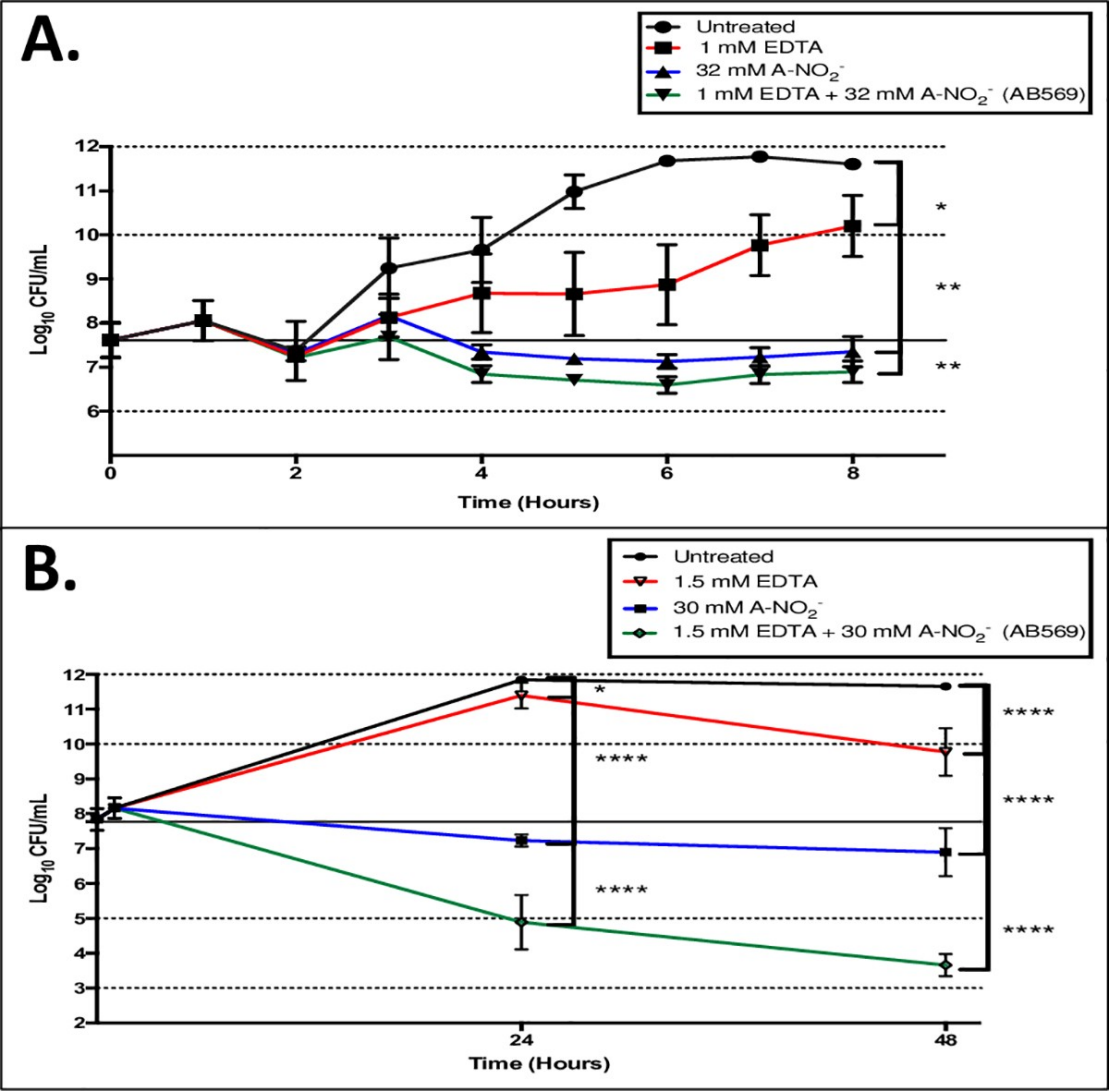
AB569 killing study *Ab* 19606. CFU counts transformed to Log10 and plotted in semi-log scale. Black line represents untreated control values, red lines are EDTA only samples, blue lines are A-NO_2_^-^ only, and green lines are EDTA + A-NO_2_^-^ (AB569) combination. **(A)**. Log_10_ CFU/ml counts with 8-hr exposure time and treated with 1 mM EDTA only, 32 mM A-NO_2_^-^ only, and 1 mM EDTA + 32 mM A-NO_2_^-^ AB569 combination, n = 3. **(B)**. Log_10_ CFU/ml counts with 48-hr exposure time and treated with 1.5 mM EDTA, 30 mM A-NO_2_^-^, or 1.5 mM EDTA + 30 mM A-NO_2_^-^ AB569. Each experiment was performed at least 3 times.

Killing studies (or Time-kill kinetic tests (36)) were performed to plot a time course for *Ab* AB569 treatment and also to optimize the conditions for qPCR analysis of selected genes that may play a role in AB569 killing of *Ab* described below. After 8 hr of treating *Ab* ATCC 19606 with 1 mM EDTA or 32 mM A-NO_2_^-^ or 1 mM EDTA plus 32 mM A-NO_2_^-^, there was no significant killing (**Fig 3A**). However, our results indicated a bacteriostatic effect on *Ab* growth by 32 mM A-NO_2_ or 1 mM EDTA + 32 mM A-NO_2_^-^ treatments when compared to untreated bacteria (**Fig 3A**). The killing efficacy of either EDTA, A-NO_2_^-^ or both was further investigation by extending the incubation time to 48 hr. Our CFU analyses revealed bacterial killing of ~2 log by 1.5 mM EDTA, ~5 log by 30 mM A-NO_2_^-^ and ~9 log by 1.5 mM EDTA + 30 mM A-NO_2_^-^, respectively, when compared to untreated bacteria. These results clearly indicate that EDTA plus A-NO_2_^-^ (AB569) has far greater bactericidal activity than either the EDTA or A-NO_2_^-^ components alone (**Fig 3B**).

### Quantitative PCR (qPCR) of *Ab* gene expression with AB569 treatment

**Fig 4:**
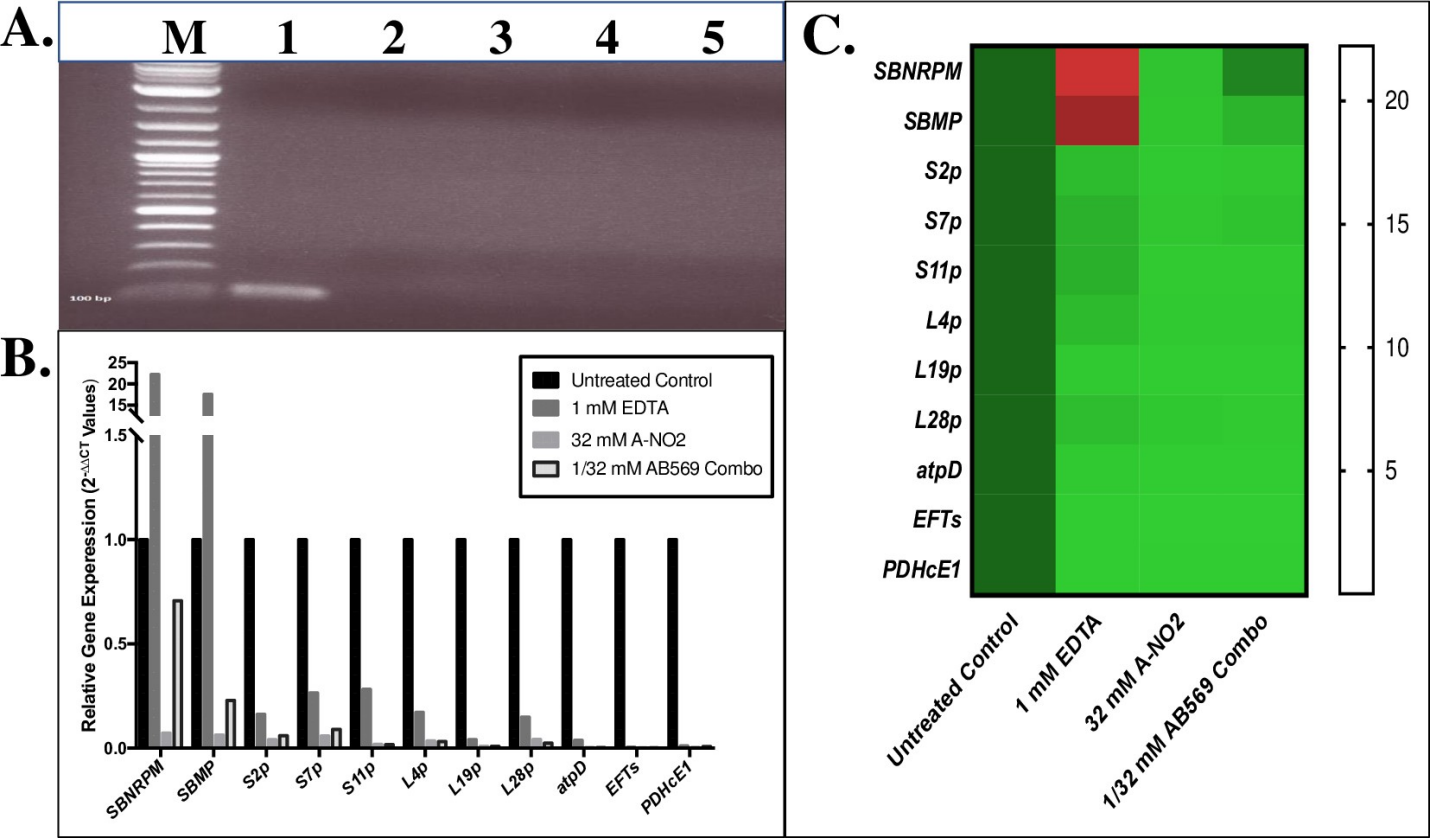
Quantitative PCR analysis of predicted genes affected by treatment of *Ab* with AB569. Total RNA Isolation following treatment with EDTA, A-NO_2_^-^, or AB569 combination (n = 3). **(A)**. Electrophoresis of purified RNA prior to RT-PCR, lane M is the ladder marker, lane 1 is genome control, lane 2 untreated control, lane 3 is sample treated with 1 mM EDTA, lane 4 is sample treated with 32 mM A-NO_2_^-^, and lane 5 is sample treated with 1 mM EDTA + 32 mM A-NO_2_^-^ AB569 combination, note the absence of dsDNA in samples compared to the positive genome control in the first lane. **(B)**. *Ab* relative gene expression (2^-ΔΔCt^ Values). Black column represents untreated control, EDTA only is dark grey, A-NO_2_^-^ only is light grey, and AB569 combination is the black outlined column. Genes tested are listed on the bottom and log scale 2^-ΔΔCt^ values are listed on the left, n = 3. **(C)**. Heat Map of qPCR 2^-ΔΔCT^ values. The genes tested are listed on the left side and treatment conditions are noted on the bottom of the graph. Gene expression is indicated by green for down regulation, red for up regulation, and black for middle range of gene expression (adjusted for contrast, n = 3).

We next delved into the preliminary mechanistic basis underlying AB569-mediated killing of *Ab* using qPCR after sublethal exposure to the drug. The genes that we predicted to be involved in this mechanism (**refer to** **Table 1**) were those that have orthologs in *Pseudomonas aeruginosa* (*Pa*), another combat wound/blast pathogen [1, 52], that we have recently shown are modulated by AB569 [32]. As both *Pa* and *Ab* are Gram-negative bacteria that cause highly problematic biofilm-based infections [53, 54], we hypothesized that the response to AB569 treatment potentially shared some common gene expression patterns. As above, the qPCR experiments were also performed using bacteria treated with EDTA, A-NO_2_^-^ or AB569. Siderophore biosynthesis non-ribosomal peptide synthetase module (*SBNRPSM*) and siderophore biosynthesis protein monooxygenase (*SBPM*) genes were first tested to evaluate the *Ab* siderophore response to the aforementioned treatments as both the EDTA and A-NO_2_^-^ components of AB569 have been recently shown to affect iron metabolism/uptake machinery in *Pa* [32]. Small subunit (SSU) ribosomal protein genes *s11p*, *s2p* and *s7p* as well as large subunit (LSU) ribosomal protein genes *L19p*, *L4p*, *L28p*, and translation elongation factor Ts (*EFTs*) genes were also selected to assess the response of these genes to the aforementioned compounds. Transcription of each of the aforementioned genes was dramatically down-regulated by EDTA, A-NO_2_^-^, and especially by AB569. The ATP synthase delta chain (*atpD*) and pyruvate dehydrogenase E1 component (*PDHcE1*) genes were selected to represent the ATP synthesis and TCA cycle transcriptional responses and these, too, showed a similar pattern of down-regulation. In contrast to the genes that were down-regulated by either treatment (**Fig 4B and 4C**), we observed a 22.2- and 17.6-fold up-regulation of the acinetobactin siderophore related genes *SBNRPSM* and *SBPM* gene transcription, respectively, in the EDTA sample only. *SBNRPSM* gene expression in the sample treated with AB569, however, was down-regulated 0.7-fold, a 0.3-fold lower expression level as compared to the untreated controls, but 0.63-fold higher than the A-NO_2_^-^ sample. *SBPM* gene expression in the AB569 sample was down-regulated 0.23-fold, 0.77-fold lower compared to the untreated control but 0.17-fold higher than the A-NO_2_^-^ sample. The EDTA samples were on average 0.16-fold higher than those of the A-NO_2_^-^ samples and AB569 treated samples in expression of *s11p*, *s2p*, *s7p*, *L4p*, and *L28p*. There was an overall downregulation compared to the untreated control with the *s11p*, *s2p* and *s7p* as well as *L4p*, and *L28p* genes. The remainder of the genes screened demonstrated decreased expression compared to the untreated control. No differences in the expression of *L19p*, *atpD*, *EFTs*, and *PDHcE1* genes were observed with either treatment.

### Effect of Ab569 on *Ab* biofilm formation and dispersion

**Fig 5:**
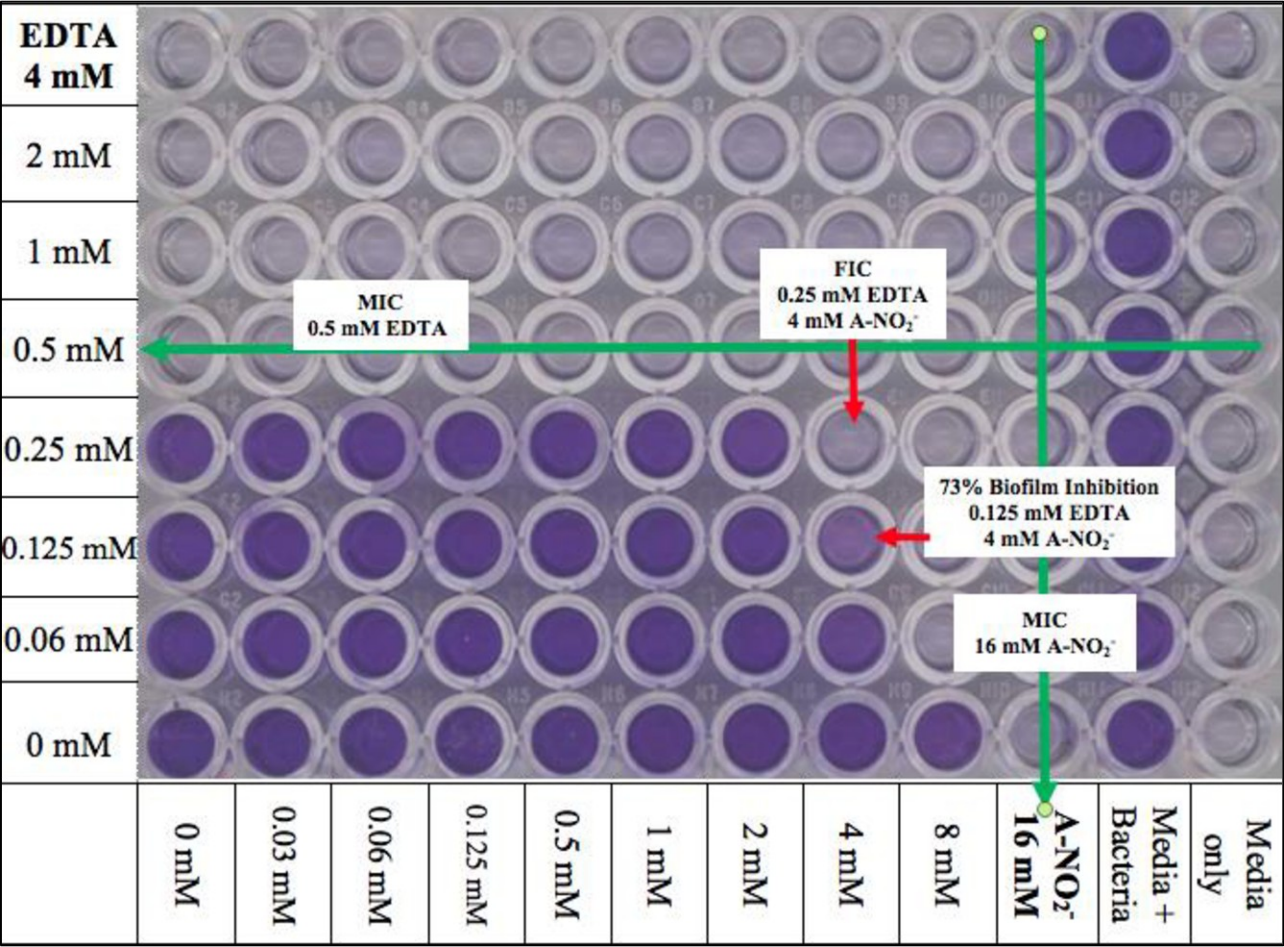
Biofilm inhibition. Scanned 96-well plate following biofilm inhibition assay. EDTA row values are indicated on the left and A-NO_2_^-^ column values are indicated on the bottom. Column 12 was the media only or background control and column 11 was media + bacteria only or the positive control. MIC values are marked with a green arrow. FIC concentrations and percent biofilm inhibition are annotated with a red arrow indicating the FIC well (n = 3).

*Ab* is an established biofilm-forming organism, a feature which also is attributed to its enhanced virulence properties [13]. The ability of *Ab* to form biofilms points to several phenotypic advantages for *Ab* pathogenesis. The production of extrapolymeric substance (EPS) is protective, as antibiotics have to penetrate the EPS to be effectively bactericidal [55]. In addition, the presence of EPS promotes increased horizontal gene transfer and antibiotic resistance in biofilm communities [56]. Therefore, the FICI of AB569 on *Ab* biofilm formation and also the effect of AB569 on biofilm dispersion was next investigated. The FIC of AB569 that inhibited *Ab* ATCC 19606 biofilm formation was 0.25 mM EDTA + 4 mM A-NO_2_^-^ while the MIC of EDTA and A-NO_2_^-^ alone to inhibit bacterial biofilm formation were 0.5 mM and 16 mM, respectively (**Fig 5**). In addition, the effect of AB569 on bacteria biofilm dispersion was also investigated. Our, results showed a 36% reduced bacterial biomass compared to untreated biofilms after treatment for 24 hr with AB569 at concentrations of 2 mM EDTA + 16 mM A-NO_2_^-^.

### Sytox Orange dead cell staining after AB569 treatment of HDFa cells

**Fig 6:**
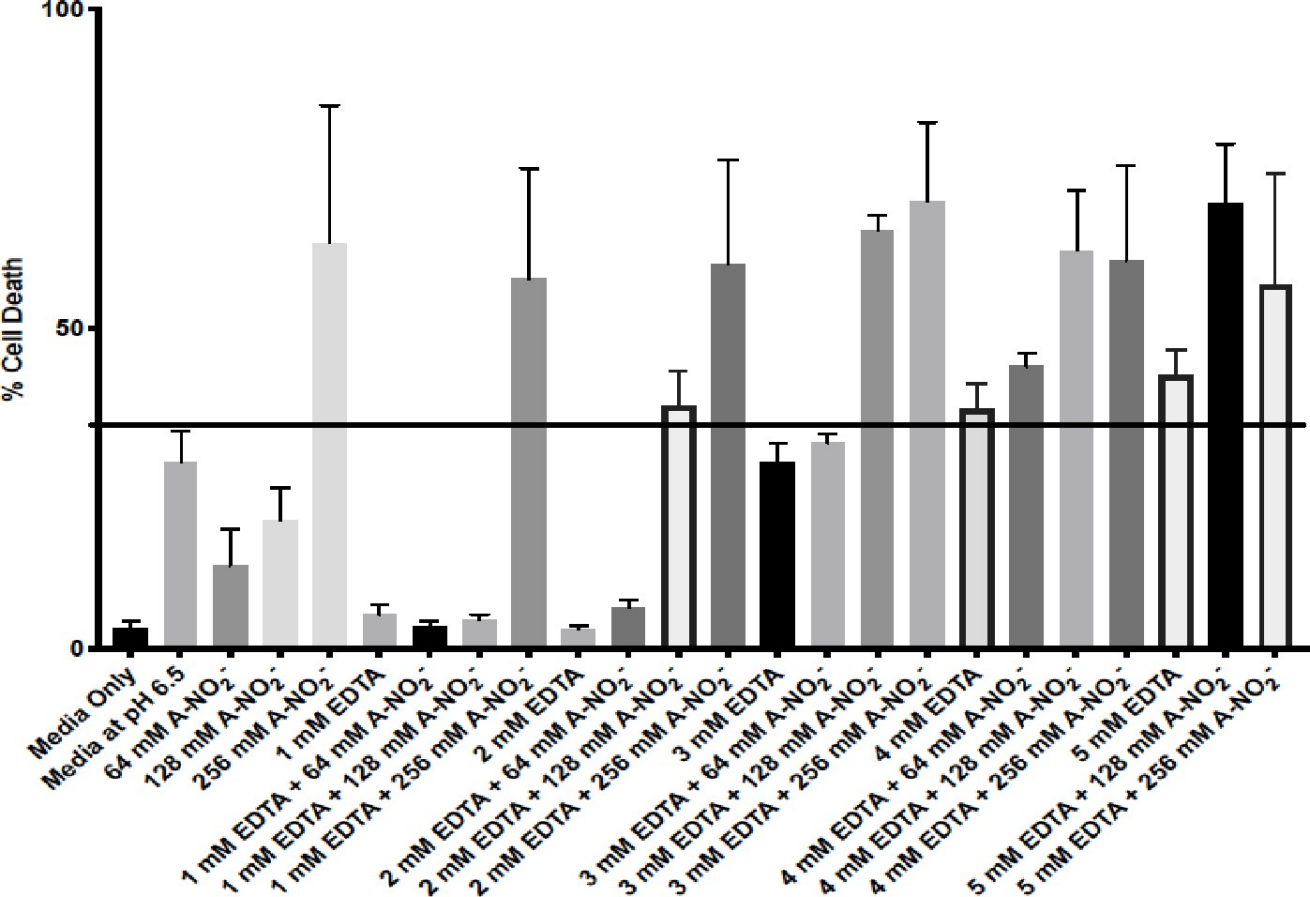
HDFa Sytox orange dead cell stain. HDFa cells treated with various concentrations of EDTA, A-NO_2_^-^ and AB569 combinations listed on the bottom axis. Each column represents a unique treatment condition. Percent cell death is listed on the left. Line indicates significant cell death at 37% compared to media only control (One way ANOVA F = 6.55, P Value = <0.0001).

To address the potential utility of using AB569 for the treatment of *Ab* infections in humans, we felt strongly that it was first critical to perform some fundamental cytotoxicity studies on skin cells. Toward this end, human dermal fibroblasts (HDFa) were used to assess the potential utility of AB569 for future wound/burn/blast treatment regiments in planned human clinical trials. Individually, it is well established that both NO_2_^-^ and EDTA have a long history of clinical use [57, 58]. A-NO_2_^-^ is used topically to treat skin infections [59], as a lung treatment for asthma [57], vasodilator [60], and ingested for its gastric anti-microbial benefits [57]. EDTA is used in chelation therapy to treat coronary artery disease [61] and in heavy metal toxicity (e.g., lead) or iron chelation treatments [11]. Predetermined bactericidal concentrations of EDTA, A-NO_2_^-^ and AB569 were used to treat HDFa cells and viability was assessed with the Sytox Orange dead cell stain and analyzed by FACS analysis. EDTA, A-NO_2_^-^ and AB569 treatment was applied at individual and combined concentrations of 1 mM to 5 mM EDTA and 64, 128, or 256 mM A-NO_2_^-^. Note that in **Fig 6**, each bar represents an individual treatment condition listed on the x-axis with the % cell death indicated on the y-axis. Media only and media (pH 6.5) controls were used to compare results and set the toxicity cut off at 32%, indicated by the black line in **Fig 6**. One Way ANOVA with Dunnett’s multiple comparisons of Sytox Orange dead cell FACs analysis revealed statistically significant treatment conditions when compared to the media only control. The combined concentrations of 3 mM EDTA and 64 mM A-NO_2_^-^ represents the highest combined concentration with insignificant cell death at a mean of ~32%. DMEM 1x supplemented media acidified to a pH of 6.5 control alone resulted in ~29% cell death.

## Discussion

This study was initiated to determine the potential utility of using a novel two-component biocide, AB569, consisting of A-NO_2_^-^ and EDTA at a pH of 6.5 to 5.5. Our primary analysis used the classical checkerboard technique [62] to measure the fractional inhibitory concentration (FIC) testing results for EDTA, A-NO_2_^-^, and AB569. The average FIC index of 0.75 revealed a non-synergistic or additive effect on *Ab* growth. Two of the reference strains demonstrated FIC values less than 0.5, an indication of synergy. These included *Acinetobacter spp genomospecies* 9, ATCC 9957, *A*. *anitratus* ATCC 49137 and *Ab* 19606T, respectively. All but one of the clinical strains demonstrated a lack of synergy; WR-1 revealed a FIC value of 0.5 with 80% antibiotic resistance. This lack of synergy differentiates *Ab* from the AB569 response in other Gram-negative bacteria including *Pa*, *E*. *coli*, *K*. *pneumoniae* and *P*. *mirabilis* [32]. *Acinetobacter spp genomospecies* 9, ATCC 9957 and *A*. *anitratus* ATCC 49137 were only 7.14% and 33.33% resistant to tested antibiotics when compared to the pronounced antibiotic resistance in the other isolates used in this study (**Table 2**).

Killing studies with AB569 demonstrated bacteriostatic activity with A-NO_2_^-^ only, while EDTA only treated samples showing little to no effect on planktonic growth with an 8 hr exposure time. The drop-in growth between the 3 and 4 hr time points in the A-NO_2_^-^ and AB569 combination samples, **Fig 3A**, demonstrates growth inhibition at shorter treatment exposure time. The inability of EDTA to kill planktonic cells was demonstrated by Lee et.al. [13] in a study that demonstrated that it played a role in *Ab* biofilm reduction. Inhibition of *Ab* growth with exposure to NO_2_^-^ was also demonstrated in a study by Weon et. al. [63] that showed the effect of NO_2_^-^ on the phosphate removal capacity of Ab in wastewater treatment. Specifically, inhibition is observed in less than 8 h of exposure. This study revealed that AB569 bactericidal activity in *Ab* treatment requires extended treatment times at concentrations that are not toxic to cells, as there was a greater than 2 log reduction in bacterial growth after 24 h of treatment.

Biofilm formation by *Ab* clearly demonstrated inhibition upon treatment with AB569. Our results demonstrated that AB569 prevented biofilm formation at treatment levels consistent with FIC results, 0.125 mM EDTA and 4 mM A-NO_2_^-^ levels for biofilm inhibition compared to the FIC results at 0.06 mM EDTA and 8 mM A-NO_2_^-^. Biofilm dispersion was at an average of 34% reduction, indicating that the tested concentrations reduced pre-formed biofilm *in vitro*. These two results together indicate that once doses are optimized for *in vivo* use (requiring costly animal toxicology studies first), AB569 may be useful as a preventative therapy for *Ab* biofilm infections or to coat surfaces used in treatment, such as indwelling catheters, tubing, ventilators or prosthetic devices.

To attempt to address the preliminary mechanism of AB569 bactericidal activity, we found significant upregulation of the siderophore-related biosynthetic genes *SBNRPSM* and *SBPM* genes in the presence of EDTA. This event may not play a direct role in killing *Ab* as the EDTA only samples did not demonstrate phenotypic killing of planktonic bacteria during AB569 killing studies. AB569, however, did demonstrate an average of 75% biofilm inhibition. Lee et.al. [13] validated the role of EDTA in reducing *Ab* biofilm formation along with McConnell et.al. [15] indicating that iron influences the amount of biofilm formed by *Ab*. This may explain the role of EDTA in the treatment of *Ab* with AB569, as increased siderophore activity indicates lower than normal iron levels during growth [14]. There is a 14-fold decrease in A-NO_2_^-^ only *SBNRPSM* gene transcription compared to the *SBNRPSM* gene transcription in the AB569 combination. Although, both were down-regulated compared to the untreated control. *SBPM* gene activity showed a similar trend to *SBNRPSM* transcription. In addition, the loss of response for siderophore production in AB569 (which still includes EDTA) could indicate a potentially important point for mechanistic studies as to why the combination is able to kill bacteria, even when not synergistic. Overall there was down-regulation with the other genes tested compared to untreated controls indicating lower ribosomal transcription levels.

**Fig 7:**
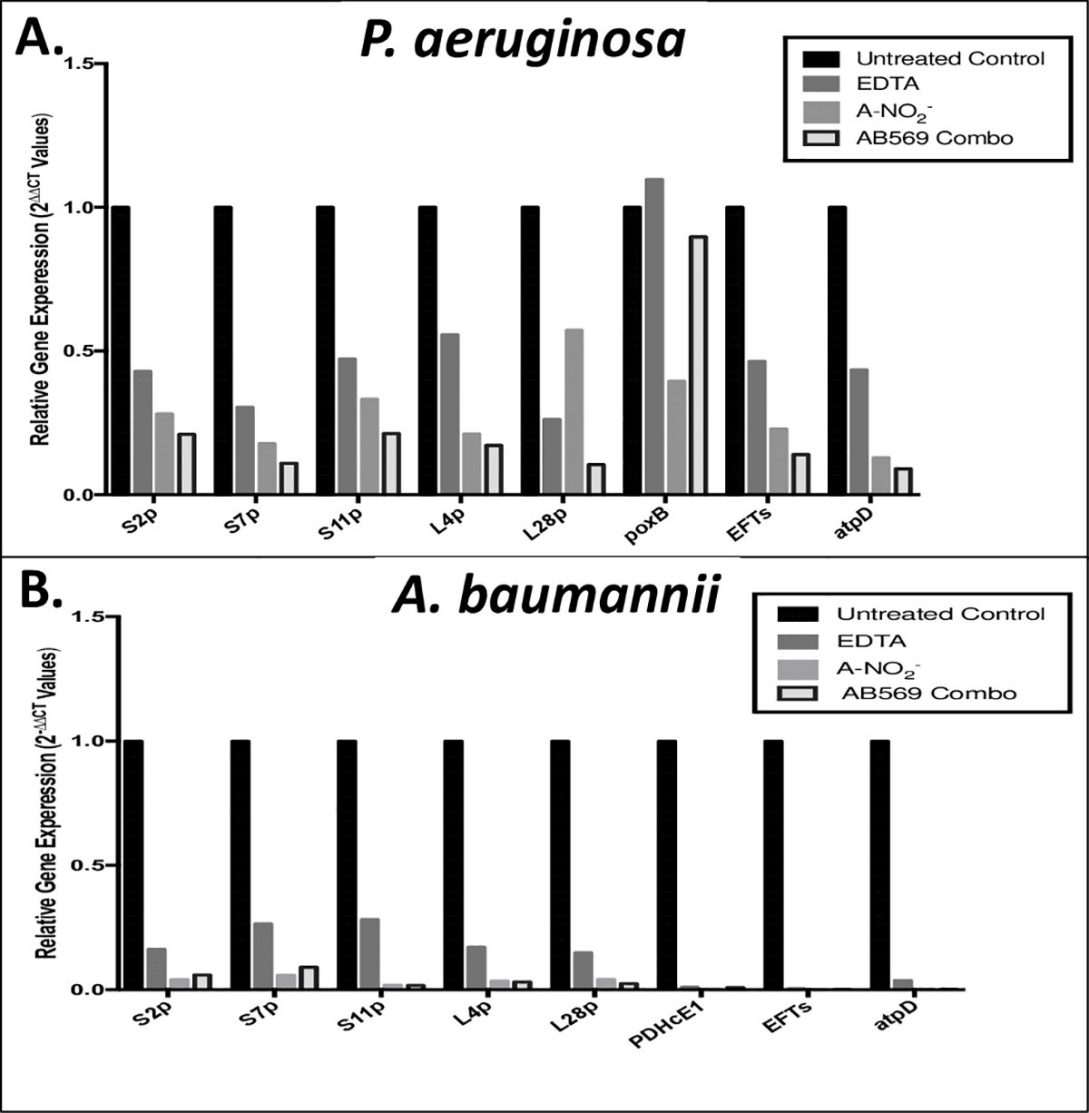
Comparison of *Pa* and *Ab* qPCR, gene expression following treatment with EDTA, A-NO2- and AB569 combination. The black column represents the untreated control, EDTA is in dark grey, A-NO_2_^-^ is in light grey, and the combination is the black outlined column 2^-ΔΔCt^ values. Genes tested are listed on the x-axis and log scale values are listed on the y-axis. **(A)**. *Pa* relative gene expression (2^-ΔΔCt^ Values). **(B)**. Ab relative gene expression (2^-ΔΔCt^ Values).

The genes selected for qPCR analysis in this study were based on previous studies with AB569 and up- and down-regulated genes in another prominent wound/blast pathogen, *Pa*. After reviewing the expression patterns in both organisms with one exception, all the genes tested in *Pa* and *Ab* showed a predicted decreased gene expression when related to untreated control with no significant difference in gene expression between individually treated EDTA and A-NO_2_^-^ compared to the AB569 combination (**Fig 7** [32]). With the exception of *poxB* in *Pa*, gene expression upon exposure to AB569 in *Pa poxB* was at ~0.9-fold while EDTA was at ~1.0-fold and A-NO_2_^-^ was at 0.39-fold expression. *Pa poxB* expression was decreased compared to untreated controls and only increased in the EDTA sample by 0.09-fold. The *Pa* gene *poxB* is pyruvate dehydrogenase (cytochrome *b*) compared to *PDHcE1* pyruvate dehydrogenase E1 component in *Ab* that enzymatically catalyzes a mechanism of activation of the required co-factor, thiamine pyrophosphate coenzyme.

Toxicity studies with HDFa cells established a workable upper limit for cell toxicity at 3 mM EDTA and 64 mM A-NO_2_^-^. This upper range is within the average FIC values for 29 *Ab* and *Acinetobacter spp*. isolates tested at 0.25 mM EDTA and 4 mM A-NO_2_^-^. However, the media at pH 6.5 control demonstrated ~29% cell death while the media only control demonstrated 3.3% cell death. This notable difference in % cell death by acidifying the cell culture media to a pH of 6.5 (necessary for optimal bactericidal activity) may be due to the use of phosphate buffer in the treatment, bicarbonate buffers in the DMEM 1x media, or the presence of 5% CO_2_ during incubation periods, as cell culture conditions are tailored to maintain an optimal pH of 7.4 (blood pH). However, in previous studies with A-NO_2_^-^, pH levels of 6.5 were tolerated in mice using a chronic lung infection model [21]. Treatment with AB569 in wound, burns, blast or ventilator-related infections and other applicable tissues is a viable therapeutic option as the treatment ranges are below the toxicity ranges for HDFa cells. However, it should be clarified that a topical agent, whether in solution or cream form, should be buffered to a pH of 6.5 or 5.5 for optimal antimicrobial activity [32].

Overall combination therapy, such as AB569, typically can decrease antibiotic resistance and combining bactericidal agents with bacteriostatic agents can often be antagonistic [64]. In this study samples treated with EDTA only failed to demonstrate killing of planktonic bacteria. However, significant growth inhibition or killing was demonstrated with A-NO_2_^-^ and AB569 combination, respectively, suggesting that, in the case of *Ab* treatment AB569, is not antagonistic. The definition of bactericidal versus bacteriostatic in some cases is dependent on multiple factors to include bacterial load, test duration, bacterial targets and reduction in bacteria. From a clinical perspective, antibiotics are known to possess both bactericidal and bacteriostatic properties [65]. The AB569 combination has been recently shown to act synergistically with many Gram-negative pathogens [32] including *Pa*, *Salmonella typhimurium*, *E*. *coli*, *Klebsiella pneumoniae* and *Proteus mirabilis* [32]. As such, the potential benefit to the early growth inhibition with *Ab* is that in mixed infections it could prevent *Ab* from gaining a growth advantage while killing other pathogens.

A final and very important issue that is critical to the understanding of the mechanism underlying the bactericidal activity of AB569 against *Ab* are through extensive and costly mechanistic studies that are beyond the scope of this work. This would include those involving sophisticated (i) chemistry (biophysical/biochemical), (ii) RNA-seq/Tn-seq bioinformatics analyses, and (iii) potential but highly doubtful mechanisms of resistance. We offer some insight as to the possible mechanism based upon only recent discoveries in *P*. *aeruginosa* grown under strict anaerobic conditions, reminiscent of late-stage, chronic CF airway disease [32]. Although the mechanism of action of AB569 remains a mystery, it is clear that negative pleiotropic effects are in play. AB569 caused an overwhelming transcriptional dysregulation in *PA*, leading to a loss of vital cellular functions, events that are likely similar in *Ab*. Our current knowledge of the chemistry of A-NO_2_^-^ plus EDTA points to increasing the amount of NO, via both generation and protection of dinitrosyliron complexes (DNICs) and *S*-nitrosylated proteins. RNA-Seq analyses of *P*. *aeruginosa* with AB569 resulted in a cataclysmic loss of essential core pathways that include those involved in the biosynthesis of DNA, RNA, protein, and ATP biosynthesis, as well as Type III secretion and iron metabolism.

Future considerations include expanding killing studies to test higher treatment levels, as toxicity levels were found 3 mM EDTA and 64 mM A-NO_2_^-^ in HDFa cells. Additional human cell lines as well as toxicology studies in animals is warranted. Optimizing treatment conditions and ultimately performing RNA-seq/proteomic analyses would aid in the determination of the mechanisms of the bactericidal action of AB569. The aforementioned data, coupled with an analysis of downregulated genes that are deemed “essential” in PA [32], would increase dramatically the overall effectiveness of AB569 in clinical settings. Given the potential role of EDTA in this study with biofilm inhibition and dispersion of mature biofilms, the *Ab* siderophore acinetobactin [66] was predicted to greatly add clues to one critical component of fundamental bacterial metabolism, iron. Additional toxicity studies with keratinocytes, given the frequency of blast wounds infections associated with Ab, would support the data from this study and would. In addition, investigating the cell culture conditions to improve the percentage of cell death in the media at pH 6.5 control may provide more consistent results in future cytotoxicity assays. Finally, animal studies to test AB569 in a possible wound treatment or flush solution model will likely be considered a positive for novel treatments against naturally antibiotic-resistant bacteria that include *Ab*.

## Supporting information

S1 File (PPTX)
