# Supplementary figures and images for: AB569, a non-toxic combination of acidified nitrite and EDTA, is effective at killing the notorious Iraq/Afghanistan combat wound pathogens, multi-drug resistant Acinetobacter baumannii and Acinetobacter spp
Source: PLoS One. 2021 Mar 3;16(3):e0247513. doi: 10.1371/journal.pone.0247513 (PMC7928478; doi:10.1371/journal.pone.0247513)

## Slide 1
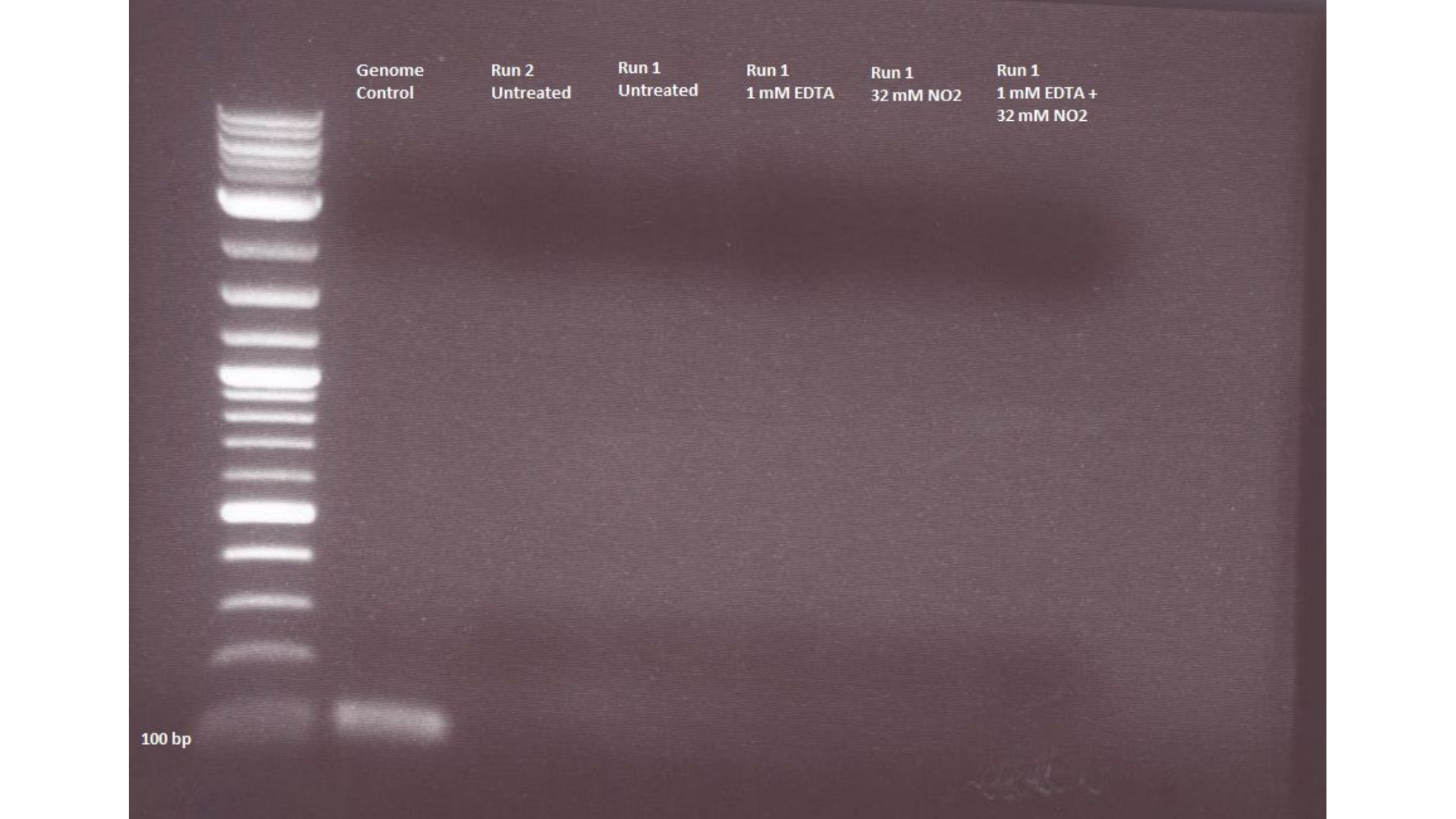

Supplement: S1 File — (PPTX) [file pone.0247513.s001.pptx]
